# Beyond the absence of sleep disorder: spotlighting the cardiovascular benefits of sleep health

**DOI:** 10.5935/1984-0063.20220002

**Published:** 2022

**Authors:** Jenny E Simon

**Affiliations:** Medical University of Warsaw, Faculty of Medicine - Warsaw - Masovian - Poland.

**Keywords:** Sleep, Cardiovascular System, Cardiovascular Diseases, Public Policy

## Abstract

Cardiovascular diseases (CVDs) are the leading cause of death globally. Among the objectives of preventive cardiology is the design to understand and neutralise the clinical overlap between disordered sleep and CVDs. Seldom do studies measure ‘sleep health’ beyond the absence of disease. Explored herein are the cardiovascular (CV) outcomes of sleep health on the grounds that, more than a corollary of sleep disorder research, sleep health constitutes a critical determinant of cardiac health and disease not unlike diet and physical activity. That sleep interventions can reverse the CV consequences of poor sleep habits lends credence to the notion that sleep health benefits CV health, and that the importance of sleep health percolates far beyond sleep disorder research. Overall, sleep health, and its practicable correlate: sleep hygiene, are clinical imperatives in the foreseeable future of cardiology in the 24-hour society.

As the leading cause of global mortality^1^, cardiovascular disease (CVD) is the object of innumerable preventive efforts. Among these has been the design to understand and harness the clinical overlap between disordered sleep and CVD^2^. A combined PubMed search of the terms sleep and cardiac yields just over 21,000 results, several hundred of which are actionable findings: clinical trials, guidelines, legislations. In the tradition of Western medicine, most of the literature is focused on the cardiovascular (CV) consequences of sleep deprivation and disorders. Fewer are the studies that measure ‘sleep health’ beyond the absence of disease. Explored herein are the CV outcomes of sleep health on the grounds that, more than a corollary of sleep disorder research, sleep health constitutes a critical determinant of cardiac health and disease not unlike diet and physical activity.

The definition of sleep health is elusive insofar as it is subject to conceptual shifts: while sleep medicine has defined itself in terms of sleep disorders and deprivation for most of its brief history^3^, modern definitions detail ‘a state of complete well-being’^4^, ‘integration’, and ‘adaptability’^5^. This abstract vocabulary can be anchored in such dimensions as sleep duration, continuity, timing, satisfaction, and daytime somnolence^6^. Sleep duration is unique in that it follows a U-shaped curve when plotted against adverse CV outcomes – hypertension^7^, coronary artery disease (CAD)^8^, arrhythmias^9^, metabolic syndrome^10^, and acute CV events such as myocardial infarction^11^ - while the other dimensions follow typical J-shaped curves. The pathophysiological mechanisms responsible for the curves’ extrema may be logically assembled as follows: Firstly, inflammation lies at the centre of the atherosclerotic process. It is thus unsurprising that the upregulation of proinflammatory cytokines interleukin 6 (IL-6) and tumour necrosis factor (TNF)^12,13^, observed in subjects with poor sleep health, is a suspected culprit of the so-called ‘residual CV risk’ in otherwise healthy individuals. Oxidative stress is the second putative mechanism with increased levels of myeloperoxidase (MPO) and insulin-like growth factor 1 (IGF-1) in subjects with poor sleep health^14,15^, both of which tip the reactive oxygen species (ROS):antioxidant scale towards atherogenesis. Importantly, a shift in nocturnal sympathovagal balance towards sympathetic predominance is reported in all subjects with poor sleep health^16^, laying fertile ground for almost every CVD. Finally, the rise in prothrombotic biomarkers such as d-dimer, von Willebrand factor (vWF), and fibrinogen is an alarming consequence of inadequate sleep^17,18^. However, the prothrombotic state remains too scarcely studied in populations with sleep disruption but without a sleep disorder to merit further mention^19^. Following the mechanistic coupling of poor sleep health to CVD is the sine qua non of this thesis: Does improved sleep predictably promote CV health?

An estimated 80% of the global CVD burden is preventable through behavioural modifications such as regular physical activity^20^. In this context, sleep health, and its practicable correlate: sleep hygiene, are clinical imperatives. That sleep interventions can reverse the CV consequences of poor sleep health was first posited by Haack et al. (2003) who reported that the blood pressure (BP)-elevating effect of short sleep duration could be corrected by means of habitual sleep extension^21^. This BP-lowering effect - a 14 mmHg beatto- beat systolic BP reduction - was evident after a 6-week period of nightly 30 minute-sleep extension and is on par with the BP reductions observed after a 6-month period of endurance training^22^ or a 9-week period of combined dietary and exercise interventions^23^. Interestingly, a 7 mmHg reduction was observed in the sleep maintenance group, likely a reflection of the use of an active control group. Indeed, subjects in this group also received a set of sleep hygiene instructions such as to ‘maintain regular bedtimes’ and ‘ensure the bedroom is quiet and dark’. While it may seem trivial at best - and medically negligent at worst - to invite ‘soft’ recommendations the likes of ‘avoid caffeine before bedtime’ into the discussion, it remains that: (a) modern society’s competing demands and ceaseless digital entertainment make sleep extension a considerable challenge for many individuals, and (b) with the notable exception of continuous positive airway pressure therapy (CPAP) in obstructive sleep apnoea (OSA), most sleep disorders are (i) underdiagnosed and -treated, as is insomnia, or (ii) inherently intractable, as in Smith-Magenis syndrome (chromosome 17p11.2 deletion syndrome). Other studies exploring the CV benefits of sleep extension found improved insulin sensitivity and loss of body fat^24,25^, hinting to the potential reversal of metabolic syndrome. More recent studies employ isotemporal substitution modeling (ISM) which examines the 24-hour behaviour of subjects in order to determine whether benefits are drawn from sustaining a fixed amount of time spent performing one activity or shifting it towards another activity. One ISM study demonstrated that replacing 91 minutes of sedentary behaviour (SB) with sleep is associated with lower waist circumference and body mass index (BMI)^26^, while another found a significantly higher CV health score after substituting as little as 30 minutes of SB by sleep^27^. CV health metrics included systolic and diastolic blood pressures, total cholesterol, fasting blood glucose, and BMI. Compounding the CV benefits of sleep health are the dietary habits that ensue. In effect, several randomised controlled trials show that balance and moderation stand in lieu of excessive appetite, overeating, and salt- and sugarrich food cravings in subjects with improved sleep health^28^. As such, measures designed to correct quantity and improve quality of sleep may compose at least part of the brake in the self-sustaining cycle between sleep deprivation and obesity, two epidemics that co-conspire in the cardiovascular disease pandemic that pervades developed and developing nations alike. Sleep deprivation mediates increases in BMI - 1 hour reduction in sleep duration per day purported to produce a 0.35 kg/m^2^ increase in BMI according to a pooled regression analysis of 634,511 adults across 30 studies^29^ - through elevated ghrelin, suppressed leptin, and increased activity of neuronal reward pathways that result in increased hunger governed by hedonic rather than homeostatic mechanisms. Further shaping the obesogenic environment are decreased levels of thyroid-stimulating hormone (TSH) and thyroxine (T^4^) that follow chronic partial sleep loss^30^ and behavioural mechanisms such as reduced physical activity due to increased fatigue and exaggerated feeding due to more time spent awake, especially irregular feeding late at night and early in the morning. Less than 5 hours (h) of sleep has been reported to increase risk of obesity by 40% compared with 7-8 h^31^, while more conservative estimates suggest a 15% and 6% increased risk in women sleeping <5 or <6 h, respectively, still in comparison with 7-8 h sleepers^32^. Obesity is, second only to cigarette smoking, a major modifiable risk factor for CVD, and, cogently put by Burman et al. (2017), sleep disturbances recently rose to prominence as obesity-related complications^33^. The link between obesity and poor sleep health is thus bidirectional with amplified convergence on CVD. Among other works whose commentary would better fit the scope of a systematic review, the aforecited findings support the notion that sleep health benefits CV health, and that the importance of sleep hygiene percolates far beyond sleep disorder research.

Burdened both by infectious diseases and a volume of CV-associated disability and death reaching pandemic proportions, a two-fold challenge exists in developing nations such that sleep hygiene guidelines are a standard that clinical practice simply cannot afford to understate. It is thereby doubly pressing that healthcare practitioners in developed countries set the example with vigorously promoted sleep hygiene guidelines. Fortunately, many sleep health schemes are well underway^34^, though admittedly thwarted by a less than responsive CV scientific community, likely a consequence of the non-uniformity of sleep specialist training across the medical specialties. Ordinarily, sleep specialists initially train in psychiatry, paediatrics, or neurology while otorhinolaryngologists are the surgical specialists with by far most ample exposure to sleep disorder patients. Meanwhile, cardiologists’ exposure ranges from little to none^35^. After all, poor sleep health is a largely modern problem, perpetuated by present-day social ills such as the 24-hour society. Globalisation has posed economic demands that oblige up to 20% of urban dwellers to work outside regular working hours, many of them older individuals within the aging workforce^36^. Similarly, the flexibility required of shift workers is intensifying as they are expected to meet the evergrowing needs of consumers of non-stop news coverage, informatics assistance, and convenience stores, among others. In keeping with Claude Bernard’s (1813-1878) conviction that the self-preservation of an organism’s biological order is critical for health and the findings of cutting-edge chronobiology research, the negative consequences of chronic sleep loss and disturbance are inevitably becoming abundantly clear. The associations between shift work and metabolic risk factors for CVD - hypertension, dyslipidaemia, insulin resistance, and obesity - are well-documented^37^, as is the increased incidence of acute CV events among this fraction of the workforce^38^. Elaborating on the disparity in sleep literacy among medical personnel, it could be speculated that nurses are more receptive to the issue given their familiarity to rotating shift work. Importantly, regardless of work hour pattern, some corporate environments may have a tendency to glorify the overworked, sleepneglectful employee prototype. Beyond chronic sleep disturbance, perhaps most evocative of the impact of poor sleep on the CV system is the finding that the risk of acute myocardial infarction increases modestly but significantly after daylight saving time (DST) transitions, that is, secondary to as little as a 1-hour change in sleep duration on the occasion of one night’s rest^39^. While it holds true that the cardiology community is increasingly active in the management of sleep disorders such as OSA, the broader incorporation of sleep medicine into daily practice, irrespective of traditional sleep disorder diagnosis, is an encouraging prospect.

Naturally, collation of literature ought to inform concrete behaviours that promote CV health. With the objective of bridging the chasm between our knowledge and CV clinical action, sleep health literacy initiatives are mushrooming^34^. Factoring in the sleep deprivation epidemic^40^ and generalised 24-hour society, poor sleep health is arguably one of the most important public health issues of our time. Clinical practice guidelines must further spotlight the aforementioned dimensions of sleep health - sleep duration, continuity, timing, satisfaction, and daytime somnolence - to overcome the ‘work hard, play hard’ attitude that leaves little room for sleep, least of all good sleep.
